# The Robust and Modulated Biomarker Network Elicited by the *Plasmodium vivax* Infection Is Mainly Mediated by the IL-6/IL-10 Axis and Is Associated with the Parasite Load

**DOI:** 10.1155/2014/318250

**Published:** 2014-03-18

**Authors:** Allyson Guimarães da Costa, Lis Ribeiro do Valle Antonelli, Pedro Augusto Carvalho Costa, João Paulo Diniz Pimentel, Nadja Pinto Garcia, Andréa Monteiro Tarragô, Maria do Perpétuo Socorro Lopes dos Santos, Paulo Afonso Nogueira, Maria Izabel Ovellar Hekcmann, Aya Sadahiro, Andréa Teixeira-Carvalho, Olindo Assis Martins-Filho, Adriana Malheiro

**Affiliations:** ^1^Programa de Pós-Graduação em Imunologia Básica e Aplicada, Universidade Federal do Amazonas (UFAM), 69077-000 Manaus, AM, Brazil; ^2^Departamento de Ensino e Pesquisa, Fundação de Hematologia e Hemoterapia do Amazonas (HEMOAM), 69050-001 Manaus, AM, Brazil; ^3^Laboratório de Imunopatologia, Centro de Pesquisas René Rachou, Fundação Oswaldo Cruz (FIOCRUZ), 30190-002 Belo Horizonte, MG, Brazil; ^4^Centro de Pesquisas Leônidas e Maria Deane, Fundação Oswaldo Cruz (FIOCRUZ), 69057-070 Manaus, AM, Brazil; ^5^Instituto de Medicina Tropical de Coari, Fundação de Medicina Tropical Doutor Heitor Vieira Dourado (FMT-HVD), 69460-000 Coari, AM, Brazil; ^6^Laboratório de Biomarcadores de Diagnóstico e Monitoração, Centro de Pesquisas René Rachou, Fundação Oswaldo Cruz (FIOCRUZ), 30190-002 Belo Horizonte, MG, Brazil

## Abstract

*Background*. Recent studies have shown that the inflammatory process, including the biomarker production, and the intense activation of innate immune responses are greater in the malaria caused by *Plasmodium vivax* than other species. Here, we examined the levels of serum biomarkers and their interaction during acute malaria. *Material and Methods*. Blood samples were collected from *P. vivax*-infected patients at admission and from healthy donors. Levels of serum biomarkers were measured by Cytometric Bead Assay or ELISA. *Results*. *P. vivax* infection triggered the production of both inflammatory and regulatory biomarkers. Levels of IL-6, CXCL-8, IFN-**γ**, IL-5, and IL-10 were higher in *P. vivax*-infected patients than in healthy donors. On the other hand, malaria patients produced lower levels of TNF-**α**, IL-12p70, and IL-2 than healthy individuals. While the levels of IL-10 and IL-6 were found independent on the number of malaria episodes, higher levels of these cytokines were seen in patients with higher parasite load. *Conclusion*. A mixed pattern of proinflammatory and regulatory biomarkers is produced in *P. vivax* malaria. Analysis of biomarker network suggests that IL-10 and IL-6 are a robust axis in malaria patients and that this interaction seems to be associated with the parasite load.

## 1. Introduction

Malaria is caused by infection with the protozoan parasite from the gender* Plasmodium* and affects millions of individuals causing serious morbidity. Although* P. falciparum* is the main cause of deaths,* P. vivax* is probably responsible for the majority of malaria cases worldwide [1], with a range of annual infections at 132 million to 391 million [2, 3].

The exacerbation, as well as the control, of the disease depends on factors related to both host and parasite. In the murine model of malaria, proinflammatory cytokines, such as IFN-*γ* and IL-12, are required to control the parasite load in the circulation [4].* P. falciparum* experimental infection of individuals who never had malaria caused an increase in the levels of circulating proinflammatory biomarkers IFN-*γ*, IL-12p40, and CXCL-8 at the time of the appearance of parasitized erythrocytes [5]. The production of proinflammatory cytokines is also able to trigger pathology and the mechanism by which a balance is achieved is still unknown. The immunoregulatory cytokine IL-10 is also produced upon* Plasmodium* infection, and it is likely to contribute to the regulation of inflammatory responses during malaria [6]. Indeed, high levels of IFN-*γ* associated with low levels of IL-10 have been associated with the severe form of malaria caused by* P. vivax* [7]. In fact, although most of the* P. vivax* infection has been considered a benign self-limited disease, severe complications associated with this parasite have been reported worldwide [7–11].

In addition to developing an effective innate immune response against the* Plasmodium*, the regulation of the inflammatory response is important to both achieve an effective adaptive immune response and to maintain the integrity of the host. To explore the role of immunological biomarkers during the malaria caused by* P. vivax* and the relationship among them, we perform cytokines/chemokine measurements in the serum of* P. vivax*-infected patients and healthy donors to evaluate the different profiles of these biomarkers and its correlation to parasitemia.

## 2. Subjects, Materials, and Methods

### 2.1. Ethics Statement

All protocols and consent forms were approved by the Ethical Committees on Research from the Fundação de Hematologia e Hemoterapia do Amazonas (HEMOAM) through the protocol CAEE (no. 0014.0.112.000-11). A signed informed consent was obtained from each subject, according to Resolution 196/96 at the National Health Board for research involving human subjects prior to enrollment in the study.

### 2.2. Patients and Healthy Controls

In this study, we have enrolled 77* P. vivax*-infected patients recruited at the Instituto de Medicina Tropical from Coari (linked to the Municipal Health Departament of Coari and the Fundação Medicina Tropical Dr. Heitor Vieira Dourado—FMT-HVD), in Coari, a malaria endemic area in the Amazon region of Brazil. The clinical manifestations of malaria were fever, myalgia, chills, arthralgia, nausea, vomiting, or diarrhea, but no patient had severe or complicated malaria. Thirty seven healthy donors (HD), living in same endemic area, who were negative for* P. vivax* infection by thick blood film and did not have previous history of malaria, were included as a control group. All participants were submitted to a detailed serological screening, recommended to monitor blood borne pathogens by Brazilian Blood Bank Authorities that includes serological analysis using an automated chemiluminescent immunoassay for the detection of Hepatitis B Virus, hepatitis C virus, and HIV infection (ARCHITECT i2000SR Plus, Abbott Diagnostics) at the Serology Laboratory at HEMOAM. Subjects under eighteen years, pregnancy status, ongoing treatment for malaria, and patients coinfected with HIV and viral hepatitis were excluded. Clinical and demographical data were acquired through a standardized questionnaire, and the hematological profile was assessed by automated complete blood count carried out at FMT-HVD from Coari.  Table 1 summarizes epidemiological, parasitological, and hematological data of* P. vivax* infected-patients and controls.

### 2.3. Blood Collection Parasitemia Counting

The parasitemia was estimated by semiquantitative microscopy analysis of thick blood smear, as recommended by the Brazilian Ministry of Health, available at http://bvsms.saude.gov.br/bvs/publicacoes/malaria_diag_manual_final.pdf. Whole blood samples were taken directly by fingerstick, venous blood collection without anticoagulant or in EDTA. After collection, the thick blood smear was prepared and air-dried at room temperature and submitted to Giemsa staining. Parasitemia was first estimated by semiquantitative traditional method, examining under 1.000x light optical microscopy. The results were expressed as follows: +/2 for 40–60 parasites/100 fields; + for 1 parasite/field; ++ for 2–20 parasites/field; +++ for 21–200 parasites/field; and ++++ for >200 parasites/field. The semiquantitative data were further used to estimate parasitemia as follows: +/2 = 200–300 parasites/*μ*L; + = 301–500 parasites/*μ*L; ++ = 501–10,000 parasites/*μ*L; +++ = 10,001–100,000 parasites/*μ*L; and ++++>100,000 parasites/*μ*L.

### 2.4. Serum Collection, Processing, and Transport

Blood samples from patients and controls were collected in BD SST tubes Gel II Advance (BD Biosciences, San Jose, CA, USA). Afterwards, the samples were stored into insulated transport boxes for biological material and shipped to the Laboratory of Molecular Biology at the Universidade Federal do Amazonas, Campus in Coari, where the samples were centrifuged in a refrigerated centrifuge (5702R, Eppendorf, Hamburg, DEU) for 5 min. at 1900 ×g. Serum was separated into aliquots in cryotubes and immediately frozen and maintained at −80°C. All samples were processed in approximately one hour. The frozen aliquots were shipped in dry ice to the Flow Cytometry Core Facility at the HEMOAM, where the biomarker measurement was performed.

### 2.5. Serum Biomarkers Measurements

The cytokines IL-2, IL-4, IL-6, IL-10, TNF-*α*, and IFN-*γ* were measured in cryopreserved serum (at −80°C for up to 3 months) using the Cytometric Bead Array kit (CBA, BD Biosciences Pharmingen, USA) following manufacturer's instructions. The serum biomarkers IL-1*β*, IL-5, CXCL-8, and IL-12p70 were measured by ELISA (Kit Human BD OptEIA Set II, BD Biosciences Pharmingen, EUA). The limits of detection for each biomarker were provided by the manufacturer as follows: IL-1*β* = 3.9 pg/mL; IL-2 = 2.6 pg/mL; IL-4 = 4.9 pg/mL; IL-5 = 7.8 pg/mL; IL-6 = 2.4 pg/mL; CXCL-8 = 3.1 pg/mL; IL-10 = 4.5 pg/mL; IL-12p70 = 7.8 pg/mL; TNF-*α* = 3.8 pg/mL; and IFN-*γ* = 3.7 pg/mL.

### 2.6. Data Analysis

In this study, we have applied three sets of data analysis approaches, referred as (a) conventional statistical analysis, (b) analysis of biomarker signature, and (c) biomarker network interactions. The second and the third approaches are innovative/nonconventional data analysis that have been shown to be relevant to detect, with high sensitivity, putative minor changes in the biomarker signatures that are not detectable by conventional statistical approaches.

*(a) Conventional Statistical Analysis.* Statistical analysis was carried out using SPSS (version 13.0). Data normality was confirmed by Shapiro-Wilk test. For continuous variables with normal distribution, comparisons of mean values between two groups were performed by Student's *t*-test. Statistical analysis among three or more groups was performed by ANOVA, followed by Tukey posttest to compare pairs. For continuous variables with nonnormal distribution, comparisons of median values between two groups were performed by Mann-Whitney test. For comparisons among three or more groups, data analysis was performed by Kruskal-Wallis, followed by Dunns posttest to compare pairs. *P* values < 0.05 were considered statistically significant.

*(b) Analysis of Biomarker Signature.* The biomarker signature was assembled as previously reported by Luiza-Silva and coworkers [12]. Briefly, the “global median” value for each biomarker was calculated taking the whole universe of data (HD + MAL). The “global median” for each biomarker was used as the cutoff to tag each individual as they display “Low” or “High” levels serum biomarkers as follows: IL-1*β* = 0.00; IL-2 = 3.69; IL-4 = 8.54; IL-5 = 3.56; IL-6 = 17.68; CXCL-8 = 6.25; IL-10 = 21.61; IL-12p70 = 988.40; TNF-*α* = 6.83; IFN-*γ* = 3.34. This strategy allowed for final computation of the percentage of individuals displaying “High” cytokines levels. Afterwards, the “ascendant biomarker signature” for the HD group was then assembled and taken as the “reference curve” (-■-) to highlight the changes in the cytokine profile of MAL patients. Relevant frequencies were considered when over 50% of the study group was confined into the “higher producers.”

*(c) Biomarker Network Interactions.* After performing the correlation analysis between biomarkers, a database was created. We then used the software Cytoscape 2.8.3 (link http://www.cytoscape.org/download.php) to assess the interactions between the correlations found. The analyses were done according to the software instructions. The thickness of the lines was adjusted to represent the strength and the kind of the correlations, positive or negative. Filled or opened circles were used to represent high and low production of which biomarker, respectively. The biomarker networks were constructed using four layouts, one for each group (HD and MAL) and subgroups studied (low parasitemia and high parasitemia). Connecting edges underscore negative, moderate, or strong correlations. Moreover, “preserved” and “malaria-related” as well as “malaria-lost” connections were also evaluated.

## 3. Results

### 3.1. *P. vivax* Malaria Patients Display High Levels of Serum Biomarkers

**Figure 1:**
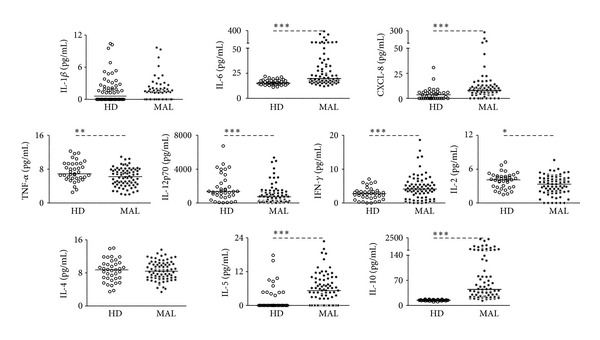
High levels of IL-6, CXCL-8, IFN-*γ*, IL-5, and IL-10 are found in the circulation of patients infected with* P. vivax*. The biomarkers IL-1*β*, IL-6, CXCL-8, TNF-*α*, IL-12p70, IFN-*γ*, IL-2, IL-4, IL-5, and IL-10 were measured in the serum of healthy donors (open circles) and* P. vivax*-infected subjects (closed circles). Levels of biomarkers were measured employing ELISA or Cytometric Bead Array (CBA).

High levels of the proinflammatory biomarkers, IL-6, CXCL-8, and IFN-*γ*, were found in serum from* P. vivax*-infected patients, when compared to healthy donors (HD). The levels of IL-5 and IL-10, regulatory cytokines, were also higher in* P. vivax*-infected patients than in healthy donors (Figure 1). The levels of TNF-*α*, IL-12p70, and IL-2 were lower during infection. The levels of IL-1*β* and IL-4 in the serum did not differ significantly when patients were compared with HD (Figure 1).

### 3.2. The Production of IL-6 and IL-10 Is Associated with the Levels of Circulating* P. vivax*

**Figure 2:**
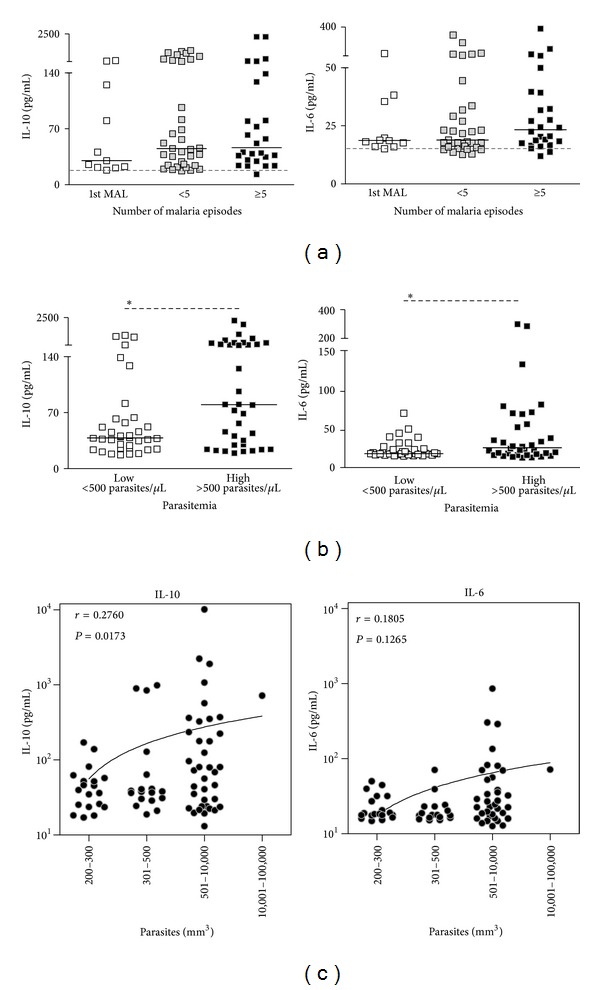
Levels of IL-6 and IL-10 are associated with parasitemia. Levels of IL-6 (figures on the right) and IL-10 (figuress on the left) were measured and analyzed based on the number of malaria episodes (a) and on the parasitemia (b). Correlation between the levels of IL-6 and IL-10 (*y*-axis) and parasite load (*x*-axis) (c). Levels of biomarkers were measured employing ELISA or Cytometric Bead Array (CBA).

The levels of IL-6 and IL-10 were evaluated in patients presenting the first episode of malaria, patients reporting less than five or more episodes of the disease. Similar levels of both IL-6 and IL-10 were seen in the three groups of patients analyzed (Figure 2(a)). Although no relationship was observed between the number of malaria episodes and the levels of IL-6 and IL-10, patients with higher parasitemia produced higher levels of both cytokines (Figures 2(b) and 2(c)). Correlation analysis demonstrates that despite the absence of correlation between IL-6 and parasitemia, a correlation between IL-10 and parasitemia levels was observed (Figure 2(c)). No correlations were observed between the other biomarkers assessed and the number of malaria episodes or parasitemia (data not shown).

### 3.3. Overall Signature of High Biomarker Producers Triggered by* P. vivax* Infection

**Figure 3:**
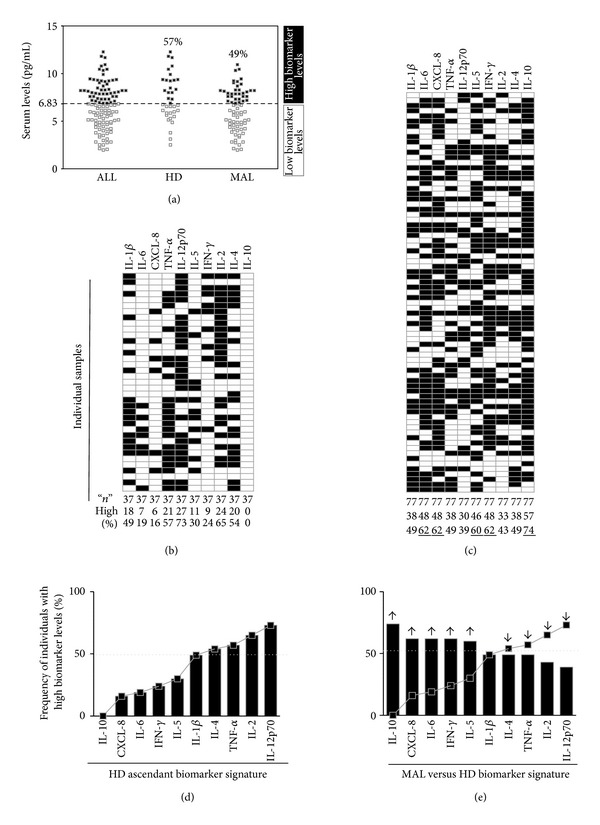
Overall signature of high biomarker producers triggered by* P. vivax* infection. (a) Representative scheme for global median cut-off calculation, using serum TNF-*α* levels as an example to categorize the individuals as those presenting “low” (white boxes) or “high” (black boxes) biomarker levels. Gray-scale diagrams were used to compute the frequency of individuals with high biomarker levels into healthy donors—HD group (b), and* P. vivax*-infected patients—MAL group (c). Bars graphs represent the proportions of individuals with high biomarker levels of HD and MAL groups (d and e, resp.). The HD ascendant biomarker profile was converted in a reference line overlaid for comparative analysis with MAL. Relevant differences were considered when the frequency of individuals with high biomarker levels in the MAL groups shifted to a distinct 50th percentile side (dotted line).

Aiming to further characterize the overall signature of serum biomarkers, the global median for each biomarker was calculated for* P. vivax*-infected patients and HD as described in Section 2. Patients and HD who presented higher frequencies of serum biomarkers than the median were considered high producer and the ones who presented lower frequencies than the median were considered low producers (Figure 3(a)). These cutoff values were used for each individual at HD (Figure 3(b)) and MAL group (Figure 3(c)) and represented as high (black boxes) or low producers (open boxes). Proportions of individuals producing high levels of biomarkers were calculated for HD and the biomarkers were represented as an ascendant reference curve (Figure 3(d)). The HD ascendant biomarker profile was converted in a reference line overlaid for comparative analysis with MAL (Figure 3(e)). Relevant differences were considered when the frequency of individuals with high biomarker levels in the MAL groups shifted to a distinct 50th percentile side (dotted line).* P. vivax*-infected patients displayed higher frequencies of high producers of IL-10, CXCL-8, IL-6, IFN-*γ*, and IL-5 and lower proportions of TNF-*α*, IL-2, and IL-12p70 when compared with HD.

### 3.4. *P. vivax* Infection Triggers a Robust Network Connecting Proinflammatory and Regulatory Biomarkers

**Figure 4:**
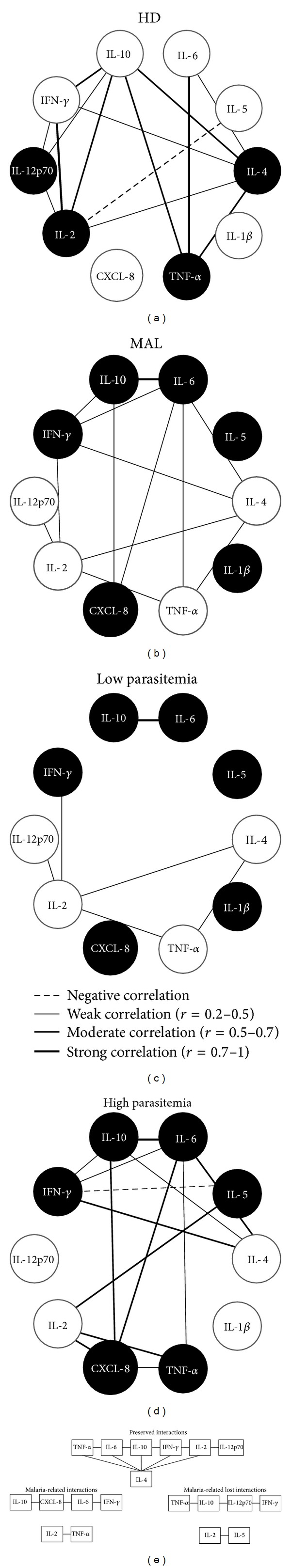
Interactions among serum biomarkers. Plasma levels of IL-1*β*, IL-6, CXCL-8, TNF-*α*, IL-12p70, IFN-*γ*, IL-2, IL-4, IL-5, and IL-10 were measured in the serum of healthy donors and* P. vivax*-infected subjects. Each connecting line represents a significant correlation between a pair of biomarkers. Dashed lines represent negative correlations. Solid lines represent positive correlations, and the degree of significance is represented by the line thickness. Filled circles represent higher producers and open circles represent low producers of a specific biomarker. (a) and (b) represent biomarker interactions in HD (a) and* P. vivax*-infected subjects (b). (c) and (d) represent biomarker interaction in* P. vivax* presenting low (low) and high parasitemia (right). (e) represents biomarkers interactions that are preserved upon* P. vivax* infection and interactions gained or lost during malaria. Circle layouts underscore each biomarker by globular nodes (° or •, as the comprise <50% or >50% frequencies of individuals with high serum levels, resp.). Spearman “*r*” indexes were used to classify the connecting edges as negative, moderate, or strong positive correlation, as shown in the figure.

In order to test in which degree the relationship among the levels of biomarker is altered upon* P. vivax* infection, a series of correlation analysis were performed. Comparisons that demonstrated significant negative or positive correlations are represented in Figure 4. The omission of a given correlation indicates that a given pair showed no associations between one another. In the absence of* P. vivax* infection, several correlations are observed. IL-6 is strongly correlated with TNF-*α* and IFN-*γ* with IL-2 (Figure 4(a)). Moderated correlations are observed between IL-10 and IFN-*γ*, IL-2, TNF-*α*, and IL-4 and between TNF-*α* and IL-4. Interestingly, besides the correlation between TNF-*α* and IL-4, all the moderate correlations are lost upon* P. vivax* infection (Figure 4(b)). A weak positive correlation was seen between IL-10 and IL-6 when the MAL was considered as single group. However, correlations indexes shifted when the analysis was performed categorizing the malaria patients in two different groups: low and high parasitemia. Correlations between biomarkers considered weak in MAL group became moderate when only patients with high parasitemia were considered (Figure 4(d)). Moreover, small correlation links between serum biomarkers were seen in patients presenting low parasite load (Figure 4(c)).  Figure 4(e) shows interactions that are malaria independent and the ones acquired and lost upon* P. vivax* infection.

## 4. Discussion

Malaria is a complex disease that affects approximately 300 million people every year, with the* Plasmodium vivax* the most geographically widespread species of* Plasmodium* causing human disease [13, 14]. While they are also essential for limiting the parasite load, in almost every infection, proinflammatory biomarkers create a harmful microenvironment in the host, leading to pathology. Upon an infection, the inflammatory response needs to modulate not only to avoid pathology but also to allow the parasite control. This delicate balance between proinflammatory and anti-inflammatory responses appears to be a major determinant of the clinical outcome of* Plasmodium* infection. Indeed, it has been reported that in severe disease caused by* P. vivax*, patients produce higher levels of IFN-*γ* than IL-10, as determined by the ratio between these cytokines [7]. On the other hand, lower ratios of IFN-*γ* and IL-10 were observed in patients experiencing the mild clinical form of the disease [7]. In the present report, we have addressed this issue by assessing the levels of immunological biomarkers and correlating them with parasitemia and number of malaria episodes. Our findings establish a clear interaction between an inflammatory cytokine, IL-6, and an anti-inflammatory cytokine, IL-10, and correlation of the former with parasite load during malaria caused by* P. vivax*.

As described here the infection with the* P. vivax* caused an increase in the levels of biomarkers produced by cells from the innate and adaptive immune system.* P. vivax*-infected patients displayed higher levels of IL-6, CXCL-8, IL-10, IFN-*γ*, and IL-5 compared to HD. The importance of IL-6 and CXCL-8 has been shown in malaria, which are produced mostly by monocytes [15]. Although previous data have reported monocytes as the main source of IL-10 among cells from the innate immune system during malaria [15], this immunomodulatory cytokine can also be produced by T cells subpopulation such as T regulatory cells (Treg) in* P. falciparum* infection [6]. Also during malaria caused by* P. falciparum*, IFN-*γ* can be produced by cells from both innate and adaptive immune system NK cells, gamma-delta T cells, and CD4^+^ and CD8^+^ alpha-beta T cells [16].

The levels of TNF-*α*, IL-12p70, and IL-2 were lower in* P. vivax*-infected patients compared to HD. Data from the literature are still unclear about the induction of TNF-*α* during* P. vivax* infection. Although studies have described higher levels of circulating TNF-*α* in* P. vivax*-infected patients, other different groups reported no induction of this cytokines during malaria [7, 15, 17–19]. TNF levels closely correlate with the temperature of patients during paroxysms and with parasitemia as well [19, 20]. In our study, IL-12p70 was lower in* P. vivax*-infected patients than in HD. Although in mice and nonhuman primates IL-12 treatment can protect against preerythrocytic malaria infections [21–23], recent human study carried out in Rondonia, Brazil [24], has demonstrated that even patients with symptomatic malaria displayed high levels of IL-12p70 as compared to healthy controls. The factors underlying the discrepancy between our data and previous reports are still unknown. It is possible that environmental and epidemiological features of distinct endemic areas as well as genetic background traits of parasites and host may account to these findings.

IL-2 has been also shown to be important during immune responses against* P. falciparum*. IL-2 leads to the activation of NK cells that in their turn produce IFN-*γ*, the most important cytokine for parasite control [4, 25].

Recently, it has been reported that the number of malaria infections caused by* P. falciparum* does not alter the levels of IL-6 and IL-10 [26]. Even in murine models of malaria, the influence of repeated exposures to* Plasmodium sp* in the production of biomarkers is not well characterized. Furthermore, repeated malaria episodes have not been able to alter significantly* Plasmodium*-specific antibody levels [27–31]. To assess the influence of repeated exposures to* P. vivax*, our patients were classified in three groups according to the numbers of malaria episodes reported. No significant differences in the levels of any biomarker were found among patients presenting the first episode, up to five or more than five episodes of malaria. The lack of difference in the levels of IL-6 and CXCL-8 according to the number of malaria episodes was expected since they are biomarkers produced by cells from the innate immune system [15, 32, 33]. However, it was expected that the levels of IL-10, IFN-*γ*, and even IL-5 presented differences between individual experiencing different number of malaria episodes due to the fact that these cytokines are also produced by cells from the adaptive immune response [15, 34–36]. Interestingly, although no relations were observed between the number of malaria episodes and the levels of IL-6 and IL-10, patients with higher parasitemia produced higher levels of both cytokines. It has been reported that* P. vivax* infection induces a significant augment of circulating IL-10 producing Treg [37]. Although no correlations were assessed between IL-10 and parasite load in this study, the authors found a positive correlation between the number of Treg and parasite burden [37].

In the absence of immunoregulation, the increase of proinflammatory biomarkers such as IL-6, CXCL-8, and IFN-*γ* can be harmful to the host. IL-6 is produced by monocytes and can act directly on monocytes/macrophages determining their fate [38, 39]. This inflammatory biomarker displays a direct association with the generation of fever, the hallmark of the acute phase of malaria [40, 41]. Although higher levels of CXCL-8, another inflammatory biomarker, are found in the circulation of* P. vivax*-infected patients, the chemotaxis of neutrophils towards this chemokine has been reported to be impaired during malaria [15]. No direct correlations were reported between CXCL-8 levels and severity or complications of malaria caused by* P. vivax*. In fact, a negative correlation between CXCL-8 levels and parasite burden was previously observed in Turkey [42]. It has been reported that mortality of patients with malaria caused by* P. falciparum* and bacteremia is three to eight times higher than that in individuals with malaria alone [43], and epidemiological evidence supports the hypothesis that infection with malaria predisposes to infection and mortality from pathogenic bacterial infections. In this context, the response of neutrophils to CXCL-8 is essential for the control of bacterial infections, avoiding complications caused by coinfection with* Plasmodium*. Different from IL-6 and CXCL-8, evidences suggest that exacerbated levels of IFN-*γ* are directly correlated with the severity of the malaria caused by* P. vivax* and the more severe the symptoms are the lower levels of IL-10 are produced [7].

To determine the interaction of biomarkers triggered by* P. vivax* infection, a network analysis was applied to the data. In the physiological conditions, strong correlations between the levels of IL-6 and TNF-*α* and between the levels of IL-2 and IFN-*γ* were observed. Interestingly these interactions become weaker upon* P. vivax* infection. Malaria patients in their turn, display a strong correlation between the levels of IL-6 and IL-10. The net of interaction also makes the alterations evident in the levels of biomarkers produced by HD and patients, as shown by the colors of the circles representing each biomarker. No interactions were observed between IL-1*β* and other biomarkers neither its levels were altered during malaria. Interestingly, when the patients were segregated in individuals presenting low and high parasite burden, important changes were observed. Only few biomarker interactions were found among patients with low parasitemia. On the other hand, the correlations between biomarkers that were weaker when patients were evaluated all together became moderate when patients presenting high parasitemia were analyzed. The strong interaction observed among malaria patients was only maintained in the group of patients with high parasitemia. However, this interaction became moderate when the network was analyzed in the group of patients with low parasitemia. In fact, the higher the levels of IL-6 and IL-10 are, the more parasites are found in the patients. Since none of our patients presented severe or complicated malaria, the higher serum levels of IL-6 and IL-10 seem to suggest an attempt of IL-6 to induce mechanisms that control the parasite and IL-10, in its turn, to control the inflammation [44]. These findings suggested that IL-6 and IL-10 may have a partial involvement in regulation of immune response during malaria infection. Mendonca and coworkers recently showed correlation between the levels of IL-10 and parasite burden in patients experiencing severe malaria caused by* P. vivax* [24]. The study also shows that individuals with asymptomatic malaria displaying multiple significant interactions involving IL-4 [24]. Indeed, the correlations preserved despite the* P. vivax* infection were in their majority interacted with IL-4.

Altogether, it is shown that both inflammatory and anti-inflammatory cytokines are produced during malaria caused by* P. vivax* and the levels of IL-6 and IL-10 are dependent on the parasite load. Moreover, a series of correlation analysis showed that the production of these cytokines is strongly associated in patients bearing high parasitemia. These findings bring new information for the understanding of the immunoregulation of inflammatory response during malaria.

## Figures and Tables

**Table 1:** Study population.

Characteristics	Healthy donors HD	Malaria patients MAL
Gender, male/female	11/26	53/24
Median age, years (range)	27 (19–45)	35 (18–73)
Number of malaria episodes, *n* (%)		
1st	—	15 (19.5)
<5	—	35 (45.4)
≥5	—	27 (35.1)
Parasitemia (parasites/mm^3^), *n* (%)		
Low, ≤500	—	39 (50.6)
High, >500	—	38 (49.4)
Symptoms, *n* (%)		
Fever	—	53 (68.8)
Myalgia	—	64 (83.1)
Headache	—	71 (92.2)
Chills	—	58 (75.3)
Sweating	—	42 (54.5)
Weakness	—	67 (87.0)
Hematological records		
Hemoglobin levels g/dL, median (range)	13.1 (11.4–16.2)	13.3 (8.4–17.1)
Hematocrit %, median (range)	38.2 (32.1–46.9)	39.2 (24.0–49.9)
Red blood cells ×10^6^/mm^3^, median (range)	4.1 (3.4–5.7)	4.1 (2.6–5.3)
White blood cells ×10^3^/mm^3^, median (range)	5.9 (3.6–11.6)	4.4 (1.4–9.0)
Platelets ×10^3^/mm^3^, median (range)	242.0 (136.0–442.0)	119.0 (20.0–270.0)
